# Integrating text mining and knowledge graph to enhance biopharmaceutical process optimization

**DOI:** 10.1371/journal.pone.0339197

**Published:** 2026-01-14

**Authors:** Shovan Bhowmik, Manju Anandakrishnan, Leah Klein, Cecilia Arighi, Marisa Gioioso, Cathy Wu, Austin Brockmeier, K. Vijay-Shanker, Chuming Chen

**Affiliations:** 1 Department of Computer and Information Sciences, University of Delaware, Newark, Delaware, United States of America; 2 Center for Bioinformatics and Computational Biology, University of Delaware, Newark, Delaware, United States of America; 3 Waters Corporation, Milford, Massachusetts, United States of America; 4 Data Science Institute, University of Delaware, Newark, Delaware, United States of America; 5 Department of Electrical and Computer Engineering, University of Delaware, Newark, Delaware, United States of America; 6 Center for Biomedical and Brain Imaging, University of Delaware, Newark, Delaware, United States of America; Universita degli Studi di Siena, ITALY

## Abstract

To guarantee consistent quality of therapeutic proteins, the relationship between manufacturing process parameters and glycosylation profiles must be investigated and understood. The most important manufacturing step to investigate is the cell culture unit operation, where glycoprotein structure is highly dependent on raw materials, cell line genetics, and process control ranges. Because of the critical role glycosylation plays in certain drug mechanisms of action, the relationship between specific process inputs and glycosylation have been documented extensively. However, despite the extensive body of published work, general relationships between different cell culture conditions and glycosylation profiles remain fragmented across diverse studies, hindering systematic analysis and data-driven decision-making. To better elucidate these general relationships from published research, we introduce an innovative framework that leverages text mining and knowledge graph technologies to automatically extract, integrate, and visualize complex relationships from scientific literature, enabling actionable insights for biopharmaceutical process (bioprocess) development. Our methodology centers on the design and development of a specialized text-mining pipeline to extract and quantify relationships between cell culture conditions (raw materials, cell line genetics, and process control ranges) and glycosylation profiles from unstructured scientific literature. To enhance precision, we implement a dual normalization strategy: 1) dictionary-based concept standardization to reconcile term variants, and 2) ontological classification to organize entities into hierarchically structured categories. These curated relationships are then systematically integrated into a knowledge graph, which not only captures direct parameter-outcome associations but also reveals higher-order indirect connection through graph, providing a comprehensive view of bioprocess interactions. We present an intuitive web-based interface that enables researchers to dynamically explore and visualize complex bioprocess relationships through interactive queries. The system demonstrates robust performance with an 88% F1-score in relation extraction, effectively revealing hidden relationships between process parameters and glycan attributes. By combining scalable knowledge graph technology with interpretable analytics, our solution empowers pharmaceutical researchers to optimize therapeutic glycan profiles and accelerate manufacturing process development. This advancement represents a significant step forward in data-driven bioprocess optimization.

## Introduction

Biopharmaceutical production involves the extraction, cultivation, or semi-synthesis of therapeutic compounds derived from living biological systems. At the heart of this technology is the use of bioreactors, which are sophisticated systems designed to enable controlled biological synthesis at both laboratory and industrial scales. These systems often maintain precise environmental parameters that are critical for optimal cell growth and product formation. The optimum conditions that contribute to a high yield and desired quality of the products include physicochemical controls such as pH, temperature, dissolved oxygen levels, along with biological factors, including source materials for cell growth and metabolism, host expression systems, and parameters related to yield optimization and product quality outcomes. They are crucial not only for production efficiency but also for ensuring the safety and efficacy of biotherapeutics, a complex product whose activity is intrinsically linked to their manufacturing process.

Glycans are sugars added to proteins during a cellular process known as post-translational modification, which occurs in the endoplasmic reticulum (ER) and Golgi apparatus during biotherapeutic production. Glycan structures vary greatly depending on cell culture conditions, and specific glycoforms may profoundly impact product stability on the shelf or safety and efficacy in the clinic [1]. Several process parameters exert significant control over glycosylation patterns, with downstream consequences for therapeutic performance [2]. For instance, elevated dissolved oxygen levels reduce both glycosylation efficiency and induce cell cytotoxicity, while increased carbon dioxide concentrations correlate with diminished glycosylation and impaired cell performance. Host cell-specific glycosylation patterns introduce additional quality challenges. For example, glycoproteins expressed in CHO (Chinese Hamster Ovary) cells frequently contain N-glycolylneuraminic acid (Neu5Gc), a sialic acid composition immunogenic to humans [3]. Similarly, chimeric therapeutics like cetuximab may incorporate immunogenic alpha-Gal epitopes that trigger hypersensitivity reactions [4]. These examples highlight the essential relationship between the process control parameters and product quality attributes, underscoring the need for effective process parameter tuning to ensure consistent glycosylation profiles and mitigate immunogenic risks in biomanufacturing.

Extensive research has explored bioreactor configurations and process parameters to characterize their impact on the biological system and synthesized products [5–7]. However, these studies often investigate isolated parameters or specific product qualities, resulting in fragmented knowledge across disparate sources. This makes it challenging to systematically identify which parameters influence desired product outcomes. A centralized knowledge base of process parameters and their influence on the product quality attributes would significantly benefit researchers by enabling a comprehensive analysis of reported data. Additionally, integrating these diverse studies could reveal novel, indirect parameter-outcome connections not explicitly documented by individual experiments. Knowledge Graphs (KGs) [8–11] offer an ideal solution for this challenge because they not only assimilate heterogeneous biopharmaceutical manufacturing data but also enable the discovery of latent relationships through structured querying and inference.

A knowledge graph is a structured semantic framework that represents a specific domain as interconnected entities and relationships. In this study, we model bioreactor process parameters and product outcomes as interconnected entities within this graph architecture. KGs provide three key advantages for bioprocess research: 1) seamless integration of heterogeneous data sources into a unified representation, 2) efficient querying capabilities to trace direct parameter-outcome relationships, and 3) inference of indirect connections through graph traversal algorithms. KG can further accommodate rich attribute annotation through node properties and edge properties, enabling context-aware knowledge retrieval essential for bioprocess optimization.

While relevant data for this study may exist across multiple sources (including proprietary databases), we focus specifically on open-source scientific literature in the biopharmaceutical manufacturing domain due to its accessibility and comprehensive coverage of diverse experimental studies. Peer-reviewed scientific articles are particularly valuable as they provide a wealth of detailed information about process parameters and product quality attributes, making them an ideal foundation for knowledge extraction. However, manually extracting information from this vast corpus is impractical due to the unstructured nature of textual data and the fact that key details are scattered throughout. To overcome these limitations, we implement an automated text-mining pipeline that systematically identifies and extracts relevant concepts, including process parameters (e.g., pH, dissolved oxygen) and product quality attributes (e.g., core fucosylation, cell cycle) from unstructured text. By automating the process, this approach organizes fragmented literature into structured knowledge ready for analysis.

Text mining (TM) automates the extraction of structured information from large volumes of unstructured textual documents by identifying and analyzing concept-specific terms and their contextual relationships. This approach enables a comprehensive analysis of a larger corpus of literature. In this work, we employ TM to extract impact relationships between process parameters and product quality attributes, represented as semantic triple <Affector, Relationship, Affected> (denoted as <*h*, *r*, *t*>). These triples are then assimilated into a knowledge graph to capture a relationship between two entities, where *h* is the head entity, *t* is the tail entity, and *r* is the relationship connecting them. For example, the statement “manganese positively correlates with afucosylation” can be structured as <manganese, positively correlates with, afucosylation>, where “manganese” is the head entity, “afucosylation” is the tail entity, and “positively correlates” is the relationship. In this paper, we refer to the head entity as “Affector" and the tail entity as “Affected" to highlight their roles. This standardized representation facilitates both human interpretation and computational analysis of complex bioprocess relationships.

In some cases, different sources may present conflicting information, often due to variations in experimental setups, biological contexts, or methodological differences. Our system does not resolve these conflicts or attempt to determine which source is correct. Instead, it presents all relevant facts as they are reported in the literature. Likewise, we do not infer causality from the extracted relationships. The relationships between the “Affector" and “Affected" are extracted from the text, reflecting how connections between entities are described in the source text, but do not confirm whether those relationships are biologically causal. It is up to domain experts to apply their own judgment to interpret these findings within the appropriate context.

While numerous process parameters can affect product quality, we specifically investigated those governing glycosylation patterns, primarily due to their significant impact on the efficacy, safety, and stability of therapeutic proteins. Additionally, precise monitoring and control of glycosylation patterns are critical for meeting regulatory standards and enhancing the therapeutic effectiveness of drugs [12]. While this study focuses on glycosylation as a representative case, the approach is designed to be modular and adaptable so that it can be expanded to include other parameters and outcomes in future work.

Scientific literature often represents identical process parameters or product qualities using multiple terms, including synonyms, abbreviations and alternative spellings. For example, “Manganese” and “Mn” both refer to the same trace metal, while “Antibody-dependent Cellular Cytotoxicity,” a clinical outcome, is often abbreviated as “ADCC.” To address this variability, we implement a rigorous term standardization protocol that maps all lexical variants to canonical representations. This “Dictionary Concept" standardization is crucial for knowledge graph development, as it eliminates data redundancy by consolidating equivalent terms and ensures accurate relationship inference by properly associating all references to the same biological entity, regardless of their textual representation in the source documents.

To enable systematic knowledge organization, we developed a domain-specific ontology that categorizes terms into hierarchical classes based on their biological and process relevance. For example, “ADCC” can be classified under “clinical outcome”, while “manganese” falls under “trace metals or minerals”. This formal ontology serves three key purposes: (1) it provides a standardized classification system for extracted terms, (2) enables querying at both instance and conceptual levels, and (3) facilitates inference of higher-order relationships. For instance, text mining extracts triples such as “manganese positively correlates with galactosylation” and “copper chloride positively correlates with sialylation”. The ontology groups “manganese” and “copper chloride” under the concept of “trace metals or minerals”, allowing queries at the ontology level (e.g., identifying product outcomes influenced by trace metals or minerals). Similarly, “galactosylation” and “sialylation” are grouped under the broader concept of “glycosylation”. By leveraging ontology, we can infer higher-level relationships, such as the positive correlation between “trace metals or minerals” and “glycosylation”.

This work presents a novel methodology for discovering latent relationships in biopharmaceutical manufacturing through the integration of text-mined data into a structured knowledge graph. We demonstrate this approach by developing a prototype of a unified knowledge base tailored for the biopharmaceutical manufacturing field. While the current prototype is focused on glycosylation patterns, the system is scalable and can be expanded to incorporate additional concepts of interest while maintaining interpretability and biological relevance. The tools and resources developed in this study are open-sourced and publicly available (https://github.com/udel-biotm-lab/BioProKGTM).

The contributions of this work are as follows:

- We developed an automated text-mining pipeline specifically designed to extract and structure relationships between process parameters and product quality attributes from biopharmaceutical manufacturing literature.
- In collaboration with domain experts, we developed a domain-specific ontology and dictionary concepts to formally capture both the terminological variations and semantic relationships common to biopharmaceutical manufacturing process.
- We integrated the text-mined data with our domain-specific dictionary concepts and ontology to construct a comprehensive knowledge graph that systematically maps relationships between biopharmaceutical manufacturing process parameters and product quality outcomes.
- To facilitate intuitive exploration of the knowledge graph, we developed a web interface that enables users to query relationships in the knowledge graph and visualize both direct and inferred relationships between process parameters and product outcomes.

### Literature review

A knowledge graph is a semantic network that represents domain-specific knowledge by capturing heterogeneous relationships between entities. KGs are widely used across various fields and offer numerous benefits such as uncovering meaningful patterns within interconnected data and generating latent-space embeddings for entities and relationships [8–11]. These embeddings enable the prediction and scoring of relationships not explicitly defined in the KG. Additionally, KGs provide structured, machine-readable domain knowledge, which enhances integration with explainable machine learning (ML) systems. Beyond ML, they serve as critical resources for grounding large language models (LLMs), improving their capacity to deliver insightful, evidence-based, and trustworthy explanations.

Knowledge graph visualization tools transform structured data in a knowledge graph into interactive graphical representations, enabling intuitive exploration of relationships between entities. Several tools exist for knowledge graph visualization, including Neo4j’s native tools and complementary systems like the Neo4j Graph Data Science library [13]. However, these tools often require writing complex queries tailored to specific use cases and lack flexibility for exploring diverse relationship patterns. To address these limitations, we developed an intuitive web interface that streamlines customized query formulation and result visualization.

Zhong et al. developed a knowledge graph framework for the traditional chinese medicine (TCM) industry to upgrade quality control from perceptual to cognitive intelligence, establishing a ‘pharmaceutical industry brain’ model [14]. Complementary research highlights generative AI’s potential to enhance TCM smart manufacturing through improved knowledge graph utilization, workforce training, and supply chain optimization—accelerating development of intelligent TCM industrial systems [15].

While several text-mining tools have been developed to extract information from the text and several impact relation extraction systems have been developed in previous studies, to the best of our knowledge, we did not find any published method towards developing text-mining tools for biopharmaceutical manufacturing. Specifically, we are not aware of any work to determine the impact of process parameters on product outcomes, which play a critical role in the clinical safety and efficacy of glycan profiles, from biopharmaceutical manufacturing literature.

Various relation extraction tools have also been used earlier to extract general relationships as well as from specific domains, such as from biomedical literature. Early efforts include a support vector machine (SVM)-based method using dependency parsing for protein-protein interaction extraction [16]. Sahu et al. introduced a Bidirectional Long Short-Term Memory (Bi-LSTM) network for extracting drug-drug interactions without relying on handcrafted features [17]. An ensemble-based method combining recurrent and convolutional neural networks was developed to improve chemical-protein impact relation based on the ChemProt dataset [18]. Additionally, Yi et al. proposed a recurrent neural network with multiple attention layers, enhancing the model’s ability to focus on relevant parts of the text for improved Drug Drug Interaction classification [19]. Recent advancements have leveraged transformer-based models for the relation extraction task. For instance, Lee et al. introduced BioBERT, a domain-specific language representation model pre-trained on large-scale biomedical corpora, demonstrating significant improvements in relation extraction [20]. BiomedBERT, a model pre-trained from scratch, was designed on PubMed abstracts, which achieved state-of-the-art results on multiple biomedical natural language processing benchmarks [21].

Moreover, adapting general relation extraction methods to specific biomedical tasks can be improved by incorporating structured lexical resources such as dictionaries and domain-specific ontologies such as entity recognition and terminology standardization [22]. Additionally, ontologies such as the Disease Ontology [23] and Gene Ontology [24] have been integrated into biomedical text mining pipelines, enhancing the systematic organization of domain-specific knowledge, resolving redundancy in terminology, and facilitating semantic categorization. In particular, ontology-driven approaches have proven successful in improving the precision and recall of biomedical relation extraction, aligning unstructured literature content with structured biomedical corpora [25–27].

A number of biomedical text-mining tools have been introduced to extract specific types of impact relations from text. SemRep [28] identifies semantic relationships such as drug-disease and treatment-disorder associations using domain knowledge and linguistic rules. PKDE4J [29] extracts protein-protein interactions (PPIs), gene-disease links, and other biomedical relations through a knowledge-driven, rule-based framework. BeFree [30] focuses on gene and disease associations using rule-based syntactic and semantic analysis. RelEx [31] is designed to detect PPIs by applying dependency parsing and rule-based extraction from biomedical text.

The Extended Dependency Graph (EDG) framework, developed by members in our group, has been implemented in several TM tools for the extraction of a variety of impact relations from the literature [32–35]. For instance, MiRiaD is designed to extract associations between microRNAs and diseases from the literature. It achieved an F1 score of 89.4%. eMIND is another text-mining tool developed to identify the impact of mutations in neurodegenerative diseases, achieving a precision of 94% and a recall of 84% in detecting relevant relationships from the literature. Other tools, such as eFIP, were developed to identify the effect of phosphorylation on the substrate proteins’ interactions with other proteins from the literature. The EDG framework leverages the Charniak-Johnson Parser [36] and the Stanford conversion tool [37] to extract relations at the syntactic level as well as propagating argument information beyond syntax, such as co-reference, appositives, is_a relationships, and part-whole relationships. The EDG served as a core component of our impact relation extraction pipeline in this project.

## Materials and methods

**Fig 1:**
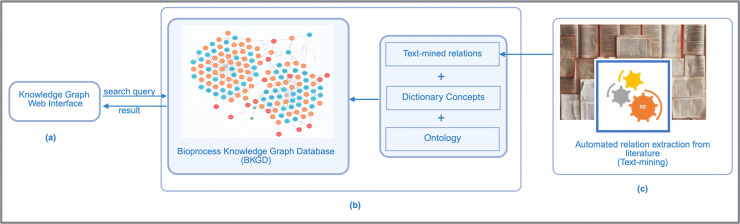
System architecture overview. (a) Knowledge graph web interface queries information from bioprocess knowledge graph database (BKGD) (b) BKGD is developed using information from text-mining, manually curated dictionary concepts and ontology (c) Automated extraction of relations from literature. Created in BioRender. Anandakrishnan, M. (2025) https://BioRender.com/e88e84c.

Our methodology comprises three core components: (1) an interactive web interface for KG exploration, (2) the Bioprocess Knowledge Graph Database (BKGD) that serves as our structured knowledge repository, and (3) a text-mining pipeline for automated knowledge extraction. Fig 1 presents the high-level architecture integrating these components, demonstrating their synergistic relationship in enabling comprehensive bioprocess analysis.

### Knowledge graph web interface

This section details the knowledge graph interface component as shown in Fig 1(a). We built an interactive web interface specifically designed to enable intuitive querying and visualization of relationships between process parameters and product outcomes. The interface provides user-friendly navigation of complex bioprocess relationships, serving as an accessible gateway to the underlying knowledge graph. The Knowledge Graph Web Interface is publicly available (https://research.bioinformatics.udel.edu/bkgd/).

#### Use cases and sample queries.

Below are examples of sample queries and use cases that can be addressed through our interface. These examples showcase the application’s utility in connecting facts from different sources. It is not intended to assign causality. In fact, when there are contradictory facts from different sources, the information is stored and displayed without any conflict resolution as the primary objective of this work is to connect the facts.

**Use Case 1:** Two independent pieces of information involving afucosylation are reported in **different sentences in an article.**

**Sentence 1**: “Using a small-scale (3 L) bioreactor model that can modulate pCO_2_ levels through modified configurations and gassing strategies, we identified three cell culture process parameters that influence afucosylation of a mAb produced by a recombinant Chinese Hamster Ovary (CHO) cell line: pCO_2_, media hold duration (at 37^∘^C), and manganese.” [PMID: 30597531] [38]

**Relation 1**: Manganese positively correlates with afucosylation.

**Sentence 2**: “The extent of afucosylation, which refers to the absence of core fucose on Fc glycans, can correlate positively with the antibody-dependent cellular cytotoxicity (ADCC) activity of a monoclonal antibody (mAb).” [PMID: 30597531] [38]

**Relation 2**: Afucosylation correlates positively with ADCC.

**Sample queries on web interface**: Does manganese affect ADCC? (or) What does manganese affect? (or) What affects ADCC? (Search for connections originating from manganese and/or ending with ADCC)

**Use Case 2**: Two independent pieces of information involving core fucosylation are reported by **different articles.**

**Sentence 1**: “As shown in Figure 6, the highest osmolality condition (500 mOsm kg^−1^ Feed C) has significantly lower levels of core fucosylation than the 410 mOsm kg^−1^ Feed C samples (*p*<0.05), despite having a lower specific antibody productivity.” [PMID: 33804825] [39]

**Relation 1**: Osmolality negatively correlates with core fucosylation.

**Sentence 2**: “Antibodies lacking core fucosylation show a significantly enhanced antibody-dependent cell-mediated cytotoxicity (ADCC) and an increased efficacy of anti-tumor activity.” [PMID: 20639190] [40]

**Relation 2**: Core fucosylation negatively correlates with antibody-dependent cell-mediated cytotoxicity.

**Sample queries on web interface**: Does osmolality affect ADCC? (Search for connections originating from osmolality and/or ending in ADCC)

**Use Case 3 (Ontology level)**: Relationships involving **two different trace metals and minerals and two glycosylation types are reported.**

**Sentence 1**: “Combinations of cytidine (Cyt), fucose (Fuc), and uridine, as well as sole addition of manganese chloride (Mn) or a mixture of Mn and galactose (Gal) with or without uridine, increased galactosylation (Figure 5a) without affecting negatively IVC or titer.” [PMID: 30552760] [41]

**Relation 1**: Manganese positively correlates with galactosylation.

**Sentence 2**: “Likewise, after the addition of compounds to increase sialylation (750 μM DANA, 50 mM ManNAc, 5 mg/ml fetuin, 0.5 mM CuCl_2_ on Day 8, and 30 μM dexamethasone), sialylated species were mainly found on the Fab fragment, and a maximum of approximately 2% Fc sialylation was observed.” [PMID: 30552760] [41]

**Relation 2**: Copper chloride positively correlates with sialylation.

**Sample queries on web interface**: What trace metals/minerals affect glycosylation? (Search for connections between trace metals/minerals and glycosylation)

#### Web interface.

**Fig 2:**
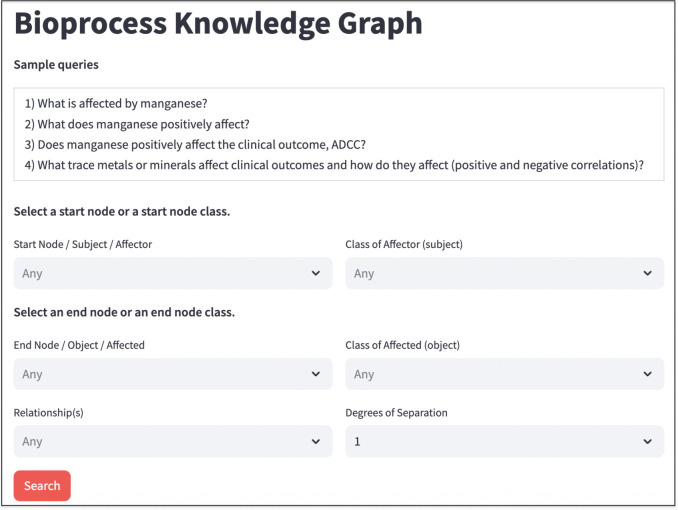
Input fields of the Knowledge Graph Web Interface.

The interface features six input fields: ‘Subject’, ‘Object’, ‘Class of the Subject’, ‘Class of the Object’, ‘Relationships’, and ‘Degree of Separation’. Users can search for paths connecting an ‘Affector’ node to an ‘Affected’ node. Fig 2 illustrates the input fields of the web interface.

Below is a detailed explanation of each field and its default values:

- Start Node/Subject/Affector: This field is populated with dictionary concepts of Affector terms from BKGD, allowing users to retrieve relationships starting from the Affector node.
- End Node/Object/Affected: This field is populated with dictionary concepts of Affected terms from BKGD, enabling users to search for relationships ending in the Affected node.
- Class of Affector (Subject) & Class of Affected (Object): These fields are populated with ontological concepts, allowing users to search for relationships at the ontology level.
- Relationship (s): This field enables users to search for one or more impact relationships of interest.
- Degree of Separation: This field defines the number of intermediate relationships between the start and end nodes. By default, it is set to 1, so direct relationships are retrieved. Users can also specify values such as < = 2, < = 3, < = 4, or < = 5 to search for transitive relationships linking the start and end nodes.

#### Query construction.

Based on the input data from the web interface, a Cypher query is dynamically generated to retrieve results from our knowledge graph using the default pattern:

“Dictionary Concept ← Affector → [Impact Relationship] → Affected → Dictionary Concept”

The dynamic query construction follows these rules:

- If the “Affector” or “Affected” field is left open-ended, a wildcard search is performed on the open-ended field using a regular expression.
- If the “Class of Affector” or “Class of Affected” is specified, dictionary concepts belonging to the selected ontological class are used as search criteria.
- The query for path traversal is expanded based on the selected “Degree of Separation” value.

#### Query results.

From the retrieved results, the properties of nodes, such as the canonical names of ‘Affector’ and ‘Affected’ terms and the properties of Impact Relationships, such as the relationship label, evidence source, and sentence, are parsed to construct triples. A triple is represented as a semantic fact in the form of <subject, relation, object>. If the results include transitive relationships, the path is expanded by linking triples together. The results are displayed in both tabular and graph formats. The graph visualization component of the interface is built using the Streamlit Agraph (https://github.com/ChrisDelClea/streamlit-agraph), a lightweight library designed for interactive graph rendering. To improve the performance of the application, some default data components are cached on the initial load.

### Knowledge graph database

In this section, we describe the component of BKGD as shown in Fig 1(b), which is hosted on a Neo4j graph database.

**Fig 3:**
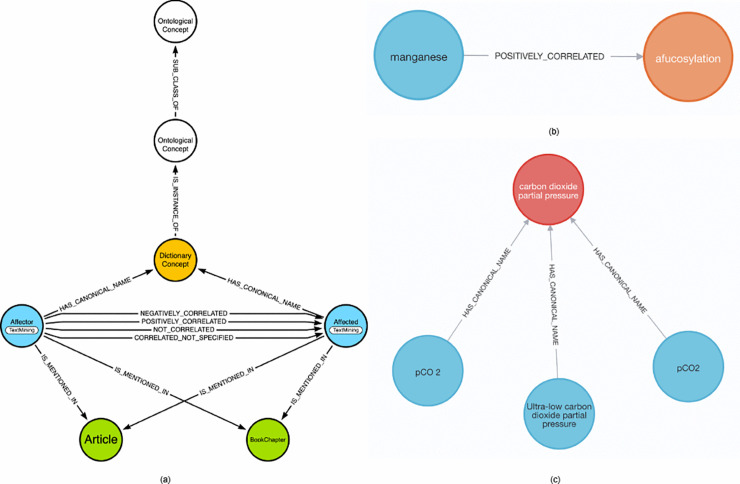
Illustration of the BKGD model with an example of an impact relationship (a) A visual representation of our graph data model showing entities and the different relationship types (b) An example relationship from BKGD showing a link between an Affector (manganese) and an Affected (afucosylation) (c) An example Dictionary Concept from BKGD with links to its variant terms.

Fig 3(a) shows the model of our graph database. Primarily, it stores the ‘Affector’ (process parameters) and ‘Affected’ (product outcomes) nodes with one of the four relationship types, positively_correlated, negatively_correlated, not_correlated, and correlated_not_specified, connecting them. Fig 3(b) shows an example of a relationship between an ‘Affector’ node, Manganese and an ‘Affected’ node, Afucosylation.

Scientific literature frequently employs multiple terminological variants to describe identical process parameters, e.g. partial carbon dioxide pressure may be mentioned as “partial pressure of CO_2_” or “pCO_2_”. To address this variability, with the assistance of subject matter experts, we created Dictionary Concepts, which maps multiple textual representations of a term to a standardized form. Without this standardization, the same term might be treated as distinct nodes, leading to fragmented connections in our knowledge graph. An abbreviation detector was used to determine abbreviations (e.g., Manganese mentioned as Mn) from the literature [42]. Fig 3(c) shows an example where varying terms representing the same concept are mapped to a standardized name.

Each dictionary concept includes the following information:

- A unique 6-digit concept ID prefixed with “COID” (automatically generated)
- Standardized name
- Alternative names (textual representations of the standardized name)
- The assigned ontological concept and its corresponding concept ID (determined by domain experts). These are stored using the properties, TYPE_ID and TYPE.

Since there is no existing work to develop an ontology for biopharmaceutical manufacturing process optimization, an ontology was designed by domain experts and features a four-level hierarchy from the highest class to the lowest subclass. For example, “nucleotide or nucleoside” is a subclass of “substrates,” which is further a subclass of “metabolic pathways”. Similarly, “serum or serum replacements” is a subclass of “substitution material,” which belongs to the broader class of “secondary raw materials”. Secondary raw materials include multiple subclasses, such as acids, carbon sources, nitrogen sources, and bases.

**Fig 4:**
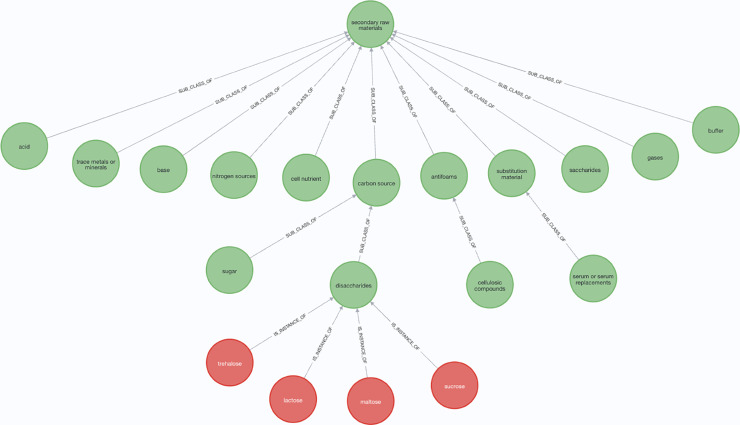
An example of hierarchical relationships in our ontology. Disaccharide is one of the lowest classes in the hierarchy of secondary raw materials. It is linked to dictionary concepts (sucrose, maltose, lactose and trehalose) with the ‘INSTANCE_OF’ relationship.

The hierarchical relationships between classes and subclasses are defined using relations such as is_a, role, catalyst, and result. For instance, the subclass “glycosylation” has an is_a relation with “PTM” (Post-Translational Modification), which in turn has an is_a relation with “biological processes”. “biological processes” further connects to the class “metabolic pathways” via an is_a relation. Conversely, the subclass “by-product or waste” is a result of the class “metabolic pathways.” The dictionary concepts connect to the lowest subclasses via an INSTANCE_OF relationship. Fig 4 illustrates an example of a hierarchy in our ontology.

The source of our knowledge graph is the relationships mined via text-mining. We used two node labels: ‘Book Chapter’ and ‘Article’, along with the relation, ‘IS_MENTIONED_IN’ to track the source of our text-mined relationships, as we processed both articles and book chapters in this work as the literature sources. Using the Neo4j Graph Data Science library and Awesome Procedures on Cypher, we transformed the text-mining data and imported it into our knowledge graph.

### Text-mining

**Fig 5:**
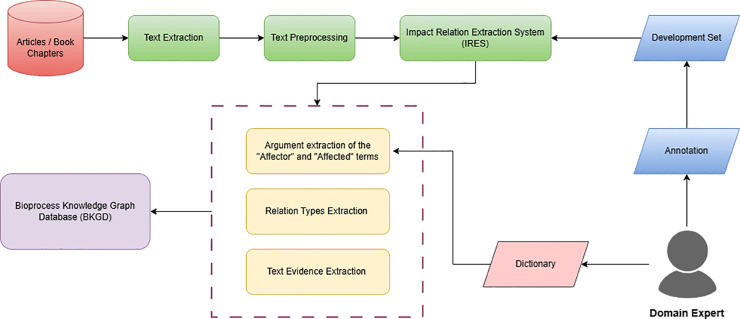
Overview of the Text-Mining pipeline showing the process of mining an article to extract relation information for the BKGD.

The main focus of our text mining (Fig 1(c)) pipeline has been on the development of an impact relation extraction system (IRES) utilizing the Extended Dependency Graph [35], an in-house framework, to extract relation arguments from the text. Fig 5 shows the step-by-step process of extracting information from literature using IRES. The text-mining workflow begins by extracting text from relevant articles and book chapters in the biopharmaceutical manufacturing domain. Next, several preprocessing steps were performed, including tokenizing the text and splitting it into sentences. The processed text is used as the input to the IRES. Using the dictionary, the extracted relation phrases containing the “Affector” and “Affected” terms were provided to the BKGD. More details of the individual steps can be found in the following subsections.

#### Text preprocessing.

The initial task in the text-mining is to extract text from the relevant articles of interest. Next, the text is split into individual sentences. For this task, “nlputils” (https://github.com/cod3licious/nlputils) library was utilized, which provides robust tools for natural language processing (NLP) tasks, including sentence boundary detection. This step ensures that each sentence is treated as a discrete unit for further analysis, enabling the relation extraction (RE) system to accurately identify and process relationships within the text.

#### Impact Relation Extraction System (IRES).

We developed the IRES to extract three components for the BKGD: i) Arguments containing “Affector” and “Affected” terms, ii) Relation types and iii) Text evidence. It was built based on a development set, curated by the domain experts after analysis of abstracts of biopharmaceutical process literature.

**Argument extraction.** Although about a hundred abstracts were annotated, it is not sufficient to induce a machine learning model for relation extraction. Hence, we adopted an in-house framework, EDG [35], to identify the arguments of the impact relation using lexico-syntactic rules.

As we moved forward to develop the system, we found that the impact relation patterns can be easily captured with common grammatical patterns of the English language, therefore, we selected the EDG approach. Additionally, we have already mentioned a few rule-based impact relation extraction tools that we developed in our earlier efforts in the literature review section. Apart from the mentioned tools, we also developed eGARD [43] (extracting genomic anomalies associated with drug response) and DiMex [44] (extracting associations between protein mutations and diseases).

The argument extraction from sentences was implemented by finding an impact relation between an “Affector” noun phrase and an “Affected” noun phrase using some grammatical rules based on the sentence structure. The impact relations could be simple impact, causal or regulation statements. The impact relation is captured by a bunch of words such as “cause”, “affect, “regulate”, “impact”, “effect”, etc. These words are called “Trigger” as they trigger the impact relation between two entities (In our case, the “Affector” and the “Affected”). We designed lexico-syntactic rules that are not only used to extract the triggers but also the two noun phrases of an impact relation. A simple rule pattern is: <Affector Noun Phrase> Trigger < Affected Noun Phrase>. For instance, our lexico-syntactic rules can extract an impact relation between the “Affector Noun Phrase” (N-linked glycosylation) and the “Affected Noun Phrase” (the potency, safety, immunogenicity, and pharmacokinetic clearance of several therapeutic proteins including monoclonal antibodies) based on the trigger word “affects” in Example 1. The “Affector Noun Phrase” is in **bold**, “Affected Noun Phrase” is in *italics* and “Trigger” is highlighted in SMALL CAPS, respectively. Likewise, the rules can also extract causal relationships between entities that also represent an impact relation. As an illustration, in Example 2, we have a rule to extract a causal relationship pattern: <Affected Noun Phrase> Trigger by <Affector Noun Phrase>. Here, the “Affector Noun Phrase” is “the more concentrated on-demand medium” and the “Affected Noun Phrase” is “the marginally higher VCD”.

**Example 1: N-linked glycosylation** AFFECTS *the potency, safety, immunogenicity, and pharmacokinetic clearance of several therapeutic proteins including monoclonal antibodies*. [PMID: 30682623] [45]

**Example 2:** This slightly higher by-product formation for the on-demand cultures resulted most likely from *the marginally higher VCD* which was CAUSED by **the more concentrated on-demand medium**. [PMID: 33656168] [46]

We also have another type of relation statement “Regulation” where the trigger is “regulate” and similar words like this. This also has a similar sentence structure as discussed above and evidence is shown in Example 3.

**Example 3:** The subsequent systematic analysis of multi-omics data showed that **pH set points** differentially REGULATED *various intracellular pathways including intracellular vesicular trafficking, cell cycle, and apoptosis*, thereby resulting in differences in specific productivity, product titer, and quality profiles. [PMID: 34289087] [47]

In our previous text-mining works [34] [35], we extracted arguments that go beyond simple impact statements as seen in the earlier part. These arguments were captured by our rules with the triggers that indicate association and do not necessarily indicate causality or regulation. In addition to using these rules for indicating association, we also identify comparison.

**Association.** Sentences can mention the impact relation between an “Affector” and an “Affected” in the form of an association. Associations between terms are indicated by the trigger words “associate”, “contribute", “involve", “correlate", etc. In particular, the word “associated” establishes an association relation between “elevated osmolality” and “an increased apoptosis rate” in Example 4. Similarly, the trigger for Example 5 is “correlate” from which we can get the relation between “The extent of afucosylation” and “the antibody-dependent cellular cytotoxicity (ADCC) activity of monoclonal antibody (mab)”.

**Example 4:** It has also been reported that **elevated osmolality** is ASSOCIATED with *an increased apoptosis rate*, although early apoptosis markers were not examined in this study. [PMID: 33804825] [39]

**Example 5: The extent of afucosylation**, which refers to the absence of core fucose on Fc glycans, can CORRELATE positively with *the antibody-dependent cellular cytotoxicity (ADCC) activity of a monoclonal antibody (mAb)*. [PMID: 30597531] [38]

It is important to mention here that the triggers in association between two entities does not explicitly indicate the “Affector” and the “Affected” terms. We decided the first argument as the “Affector” and the second argument as the “Affected” based on the rule involved in an association.

**Comparison.** We detected comparison structures in sentences because they typically indicate association. For this work, we followed our previous work [35], where we capture two compared entities and the association relation between the compared aspect and either of the compared entities. As an illustration, in Example 6, our comparison rules will provide the phrases “this increased sialylation” and “a high level of galactosylation”, from which the IRES tool will extract the association relation between sialylation and galactosylation.

**Example 6:** *This increased sialylation* was ENABLED by **a high level of galactosylation** compared to the wild-type antibody. [PMID: 25875452] [48]

**Extracting arguments from noun phrases.** The above subsections are only about the impact relations between two entities. Even if the papers are related to biopharmaceutical manufacturing, it is possible that there are mentions of entities that are not relevant to the field. Thus, our next step is to verify if the extracted “Affector” and “Affected” arguments are indeed of relevance. This is accomplished by using the ontology and the associated dictionary. For example, in Example 3, the noun phrase for the “Affected” term is “various intracellular pathways including intracellular vesicular trafficking, cell cycle, and apoptosis” from which the interested terms are vesicular trafficking, cell cycle and apoptosis. Similarly, we only need “pH” as the “Affector” term from “pH set points” to establish the relation. In fact, the EDG provides a relation between two words. Then, the IRES extracts the noun phrases that are headed by these words. As the domain experts have already developed the dictionary of relevant words, the dictionary concepts do not match the entire noun phrase. Instead, it only matches the part that is a lexicalization of the ‘Dictionary Concept’. Eventually, these matched parts are extracted from the noun phrases based on the dictionary concept as the Affector and the Affected arguments.

**Relation type extraction.** Although we got the “Affector” and “Affected” terms as discussed in the Argument Extraction subsection, it is equally important to indicate the directionality of the impact relations from the “Affector” to the “Affected”. Often, this can be obtained from the text itself. Therefore, IRES attempts to categorize the impact relation into four types: positively_correlated, negatively_correlated, not_correlated, or correlated_not_specified.

To determine the type of correlation between the “Affector" and “Affected" entities, we developed a rule-based module that leverages lexical cues from the sentence context. Specifically, we compiled lists of indicative terms to capture positive (e.g., increase, up-regulate, elevated) and negative (e.g., decrease, down-regulate, reduced) words. These cues were searched within the noun phrases corresponding to the Affector and Affected entities, as well as the trigger term connecting them.

We assigned one of the following types to each relation:

- positively_correlated: If positive terms were found in at least two components (arguments or trigger) and no negative terms were present.
- negatively_correlated: If any negative term was identified, or both positive and negative terms co-occurred in different components.
- correlated_not_specified: If none of the components contained either positive or negative terms.
- not_correlated: if a negation term (e.g., not, no, never) appeared directly preceding the trigger and suggesting the absence of a relationship.

As an illustration, the noun phrases of Example 4 have positive words (“elevated” and “increased") while the trigger does not contain any positive or negative words. Therefore, the impact relation type for Example 4 is positively_correlated.

**Example 7:** In principle, elevated ammonia levels result in **increased intracellular pH conditions** that inhibit b4GalT activity and expression, and consequently LEAD to *reduced protein galactosylation*. [PMID: 33804825] [39]

**Example 8: Cell death in bioreactors** REDUCES *productivity and product quality*, and is largely attributed to apoptosis. [PMID: 35737825] [49]

In Example 7, the “Affector Noun Phrase” (increased intracellular pH conditions) has a positive word “increased” and the “Affected Noun Phrase” (reduced protein galactosylation) has a negative word “reduced.”. The trigger word (lead) is neutral. That is why the impact relation type for Example 7 is negatively_correlated. In the same way, we have heuristics to assign the relation type based on the presence of positive or negative words associated with the arguments and the directionality of the trigger word. For instance, there is only one expressive word, “reduces” (negative), as the trigger that determines the impact between the “Affector Noun Phrase” and the “Affected Noun Phrase” which are neutral in Example 8. So, the impact relation type for this sentence is negatively_correlated.

**Example 9:** PCA was performed to assess the suitability of using MVDA to characterize the IMPACT of **media selection** on *antibody glycosylation and productivity*. [PMID: 31487120] [50]

**Example 10:** No EFFECTS of **culture pH** on *qp* could be shown in studies from Trummer et al. and Yoon et al.. [PMID: 27752770] [51]

Likewise, the positive and negative cues are not found in the “Affector" (media selection) and “Affected" (antibody glycosylation and productivity) arguments in Example 9. However, there is a trigger word “impact" between these two entities. Therefore, we assign this type of impact relation as correlated_not_specified. Example 10 provides an instance that is not_correlated because of the indicative word “No" before the trigger “effects" which signifies that the “Affector" (culture pH) does not have any correlation with the “Affected" term (qp).

These heuristics were implemented using regular expression-based pattern matching and applied to the dataset via a Python script. The full code can be found in our GitHub repository (https://github.com/udel-biotm-lab/BioProKGTM/blob/main/textmining/Scripts/EDG_Output_Process/adding_relation_types.py).

**Text evidence extraction.** In addition to determining the relation type, we also pulled the names of essential parts of an article from where each relation was extracted. As we considered three types of articles: PubMed abstracts, full-length articles, and book chapters in this research, the impact relations were extracted from various parts of the full-length articles including abstract, introduction, methods, results, discussion, etc. For abstracts, the text evidence section was straightforward as there is only one section. To identify the titles of the text evidence, we parsed the XML files of the full-length articles. For PubMed abstracts and full-length articles, we included the PMID (PubMed Identifier) and PMCID (PubMed Central Identifier) with each relation. In the case of book chapters, we extracted the chapter title. Besides, we added the ISBN (International Standard Book Number). For books, which are typically available in PDF format, we used the PyPdf2 (https://github.com/talumbau/PyPDF2) tool to divide the content into chapters and assigned the corresponding chapter number to each extracted relation. The addition of the section titles and the article identifiers of the text helps us to connect independent pieces of relational information in the knowledge graph and query interface, as discussed earlier.

#### Integration of IRES output to the BKGD.

Although earlier subsections describe each part of the IRES, it is also important to discuss the integration of the output of the IRES to the BKGD. As discussed before, we extracted the arguments of interest (“Affector”/“Affected” terms) by utilizing the dictionary concept instead of sending the whole noun phrase to the BKGD. Besides, we included other necessary information extracted through IRES and fed it to the BKGD. The final input to the BKGD had the following components: Article Identifier (PMID, PMCID, ISBN), Sentence, Affector term, Affected term, Trigger, Relation type, Text evidence, Dictionary concept id, and Ontological concepts. As noted earlier, the relationships added to the knowledge graph reflect how they appear in the text, and we do not infer causality or resolve conflicting information.

### Performance evaluation metrics for IRES

We evaluated the performance of our IRES using precision, recall, and F1-score. Precision and recall were calculated based on True Positives (TP), False Positives (FP), and False Negatives (FN), defined as follows:

- **TP:** Number of extracted relations that match the annotated relations.
- **FP:** Number of extracted relations that do not match any annotated relations.
- **FN:** Number of annotated relations that were not extracted.

To ensure fair evaluation, these metrics were also computed at the relation level, where a predicted relation was considered correct only if both entities and their relational type matched the corresponding gold-standard annotation.

Eqs 1, 2, 3 are used to calculate Precision, Recall and F1-Score respectively.

$$Precision=\frac{TP}{TP+FP}$$ (1)

$$Recall=\frac{TP}{TP+FN}$$ (2)

$$F1\text{-}score=2\times \frac{Precision\times Recall}{Precision+Recall}$$ (3)

## Results

In this section, we present the findings of this work, including tabular and graph views of example query results from our web-based interface, summary statistics of the knowledge graph, statistics of the ontological and dictionary concepts integrated into our text-mining tool, statistics of the annotated development set, evaluation performance on the annotated abstracts, and the number of extracted relations from both full-length articles and book chapters.

### Web user interface

The web user interface presents search results in both tabular and graph views.

#### Tabular view.

**Fig 6:**
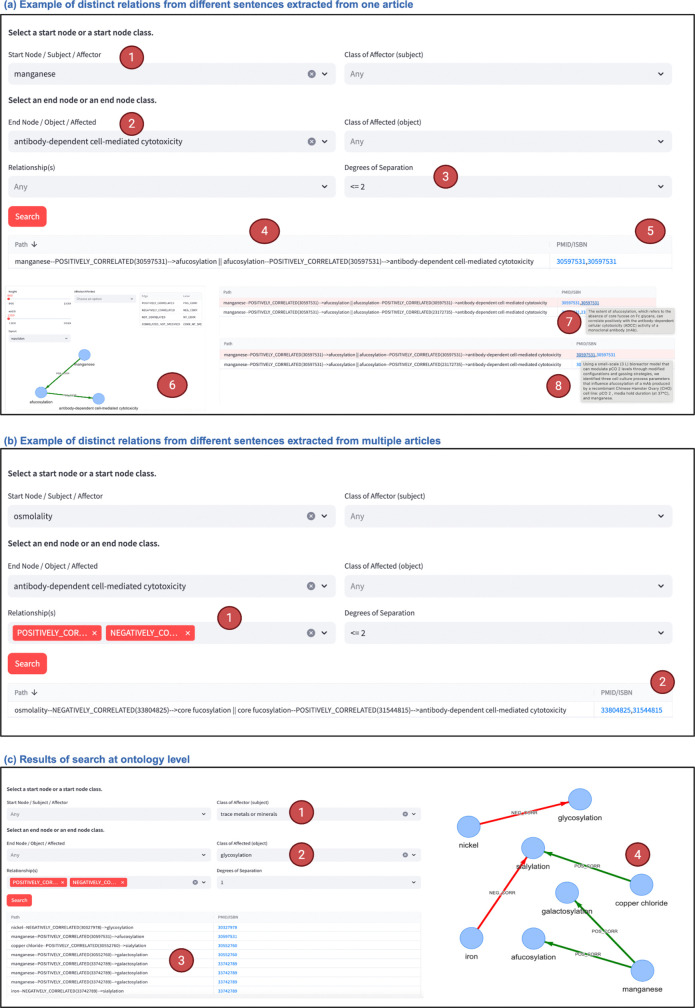
The results of three different search parameters. (a) Example where a relation from the same article and two different sentences are linked. (a.1) subject is populated with the Affector term, ‘manganese’; (a.2) object is populated with the Affected term, ‘ADCC’; (a.3) a degree of separation value ≤2 is selected; (a.4) search results show a link between manganese and ADCC via afucosylation; (a.5) the article evidence supporting the links are displayed (as both the relations are extracted from the same article, the same PMID is displayed twice); (a.6) graph view displays the connections between the entities; (a.7) On mouseover of the first instance of the PMID, the sentence supporting correlation of afucosylation and manganese is displayed; (a.8) On mouseover of the second instance, sentence source of the relation between afucosylation and ADCC is displayed. (b) Example where relations from two different articles are linked. (b.1) The impact relationship field is limited to two types – ‘Positively Correlated’ and ‘Negatively Correlated’; (b.2) the second relation (core fucosylation negatively regulates ADCC) is supported by two distinct articles and the first relation is extracted from another article. (c) Example of search result at ontology level. (c.1) ‘trace metals/minerals’ is selected as the subject class; (c.2) ‘glycosylation’ is selected as the object class; (c.3) results include multiple subject and object terms, where all the subject and object terms belong to the selected classes; (c.4) graph view provides a consolidated view of the links between the resulting entities. Created in BioRender. Anandakrishnan, M. (2025) https://BioRender.com/bh7detl.

The tabular view consists of two columns: ‘Path’ and ‘PMID/ISBN’ (Fig 6). The ‘Path’ column displays the links from the subject to the object. For transitive relationships, each triple is separated by the delimiter ‘||’, with the evidence (PMID/ISBN) for each triple enclosed in parentheses. The ‘PMID/ISBN’ column lists all supporting evidence for the link, with multiple pieces of evidence separated by commas in the order of the corresponding triples. The PMID/ISBN entries are hyperlinked, enabling users to navigate directly to the articles.

#### Graph view.

The graph configuration allows customization of components such as height, width, and layout format. It displays a graph with nodes and edges based on the search input and the specified relationship type. Users can filter the graph to show paths containing a subject or object of interest using the dropdown field ‘Affector/Affected’. Fig 6 presents the search results for three queries:

1. Fig 6**(a):** Shows results for the subject ‘manganese’ and object ‘ADCC’ with a degree of separation < = 2. This query retrieves links between manganese and ADCC mediated by afucosylation. The relationships between manganese and afucosylation, and afucosylation and ADCC, were text-mined from two sentences in an article [PMID: 30597531] [38].
2. Fig 6**(b):** Displays links between osmolality and ADCC. These relationships were extracted from two different articles. Although the information comes from distinct studies, the integrated knowledge in the BKGD enables the derivation of indirect links. Additionally, the positive correlation between core fucosylation and ADCC is supported by two separate articles.
3. Fig 6**(c):** Demonstrates a search at the ontological level, identifying trace metals/minerals that affect glycosylation. The results include “Affector" terms such as nickel, manganese, iron, and copper chloride, and affected terms such as glycosylation, galactosylation, sialylation, and afucosylation. The ontology facilitates class-level searches.

The graph view allows users to visualize the relationships between entities. Results are linked to source articles/book chapters, and hovering over a relation displays the extracted sentence.

### Bioprocess knowledge graph database statistics

**Fig 7:**
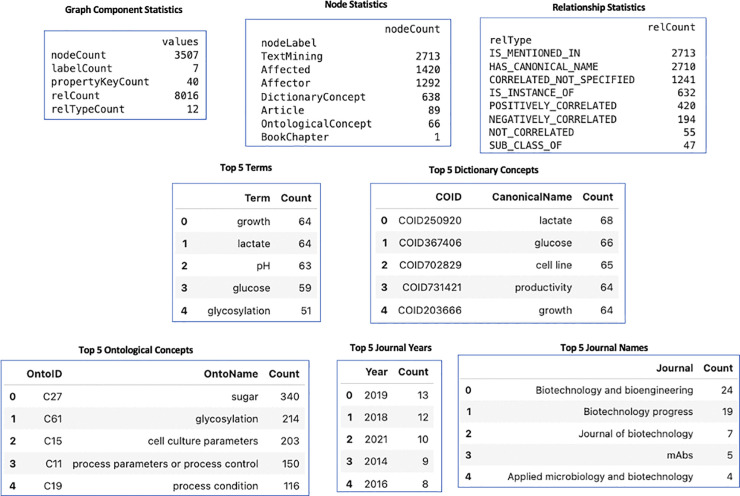
Statistics of data in our Bioprocess knowledge graph database. This includes graph component statistics, node statistics, relationship statistics, top 5 ontological concepts, top 5 dictionary concepts, top 5 terms, top 5 journal years, and top 5 journal names.

Fig 7 presents the statistics of the data in our knowledge graph database. The BKGD includes 1,292 Affector nodes and 1,420 Affected nodes, grouped under 638 Dictionary Concepts. These relationships were extracted from 89 articles and 1 book (12 chapters). The figure also highlights the most frequently occurring concepts, terms, and journals.

### Ontology

Our ontology consists of 66 classes and subclasses with 41 ontological relations. Since some classes do not have subclasses, the number of relations is fewer than the total number of classes.

### Dictionary concepts

Our tool includes 639 dictionary concepts and 1,248 terms, encompassing both canonical names and alternative names.

### Corpus construction and annotation

To adapt our general tool for domain-specific use, an annotated dataset was prepared by the subject matter experts (SMEs) following well-defined guidelines. The corpus construction process began with the SMEs providing an initial list of 95 publications with their corresponding DOI identifiers. We mapped these DOIs to PMIDs using the PMC ID Converter service at NCBI (https://pmc.ncbi.nlm.nih.gov/tools/idconv/#api) and the PMID2Cite service (https://www.pmid2cite.com/doi-to-pmid-converter).

Of these, 49 publications were used to train a model in LitSuggest [52], a web-based machine learning system for literature recommendation and curation. The trained model was then tested using the remaining publications, all of which were classified as positive, confirming the reliability of the training set.

This trained LitSuggest model was subsequently used to expand the corpus for annotation. Using the query “(mAb OR monoclonal) AND (glyco* OR glycan OR syal*) AND (CHO OR “Chinese hamster ovary" OR DG44)" since they are widely found in the biopharmaceutical process-related literature, we retrieved a set of 100 abstracts, representing both top relevant and top irrelevant publications for balanced annotation coverage. Annotations were carried out using TeamTat [53], a collaborative text annotation platform. SMEs collaboratively developed and refined the annotation guidelines. These guidelines standardized the identification and annotation process to ensure consistency and quality across annotators.

**Fig 8:**
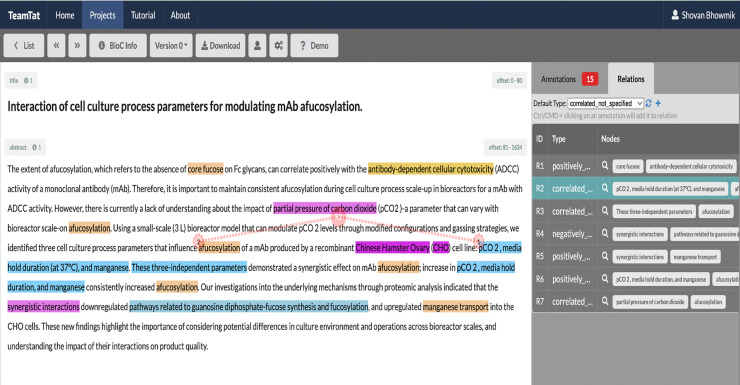
Illustration of an annotation in TeamTat. Abstracts are opened in the TeamTat interface where annotators curate sentences with impact relations. The Affector and Affected terms are marked in blue and yellow colors. The relations are established with red dotted lines. The impact relation types and the impacted arguments are also visible in the right side of the window.

Each selected abstract was reviewed to determine whether it was curatable, meaning whether it contained at least one relation between an Affector and an Affected entity. Annotators then marked up the corresponding entities and established the relational links between them. Specifically, the Affector and Affected arguments were annotated, and the type of each relationship (e.g., positively_correlated, negatively_correlated, or correlated_not_specified) was defined during the process. Fig 8 illustrates the curation of an abstract for the entities mentioned in example Sentence 1 from Use Case 1 using TeamTat.

In total, 98 abstracts were annotated for the training dataset, resulting in 445 entities and 362 relations. We used these annotated abstracts to develop the dictionary and ontological concepts, as well as build the IRES by designing its rules.

### Relation extraction

This study sought to identify relationships between biopharmaceutical process parameters and glycan quality attributes. Data were extracted from three distinct sources:

1. **Annotated Abstracts:** Served as the foundation for developing the text-mining pipeline.
2. **Full-Length Articles:** Utilized to extract relationships by applying the text-mining pipeline established from annotated abstracts.
3. **Book Chapters:** Similarly analyzed for relationship extraction following the same methodology as full-length articles.

From the 98 annotated abstracts, we extracted 354 relations out of the 362 annotated relations available. These annotated relations were exclusive to the abstracts. Additionally, we analyzed 33 full-length articles and a book [54] with 12 chapters, extracting 1,435 relations from the articles and 540 relations from the book chapters. The text-mining output was integrated into the knowledge graph to uncover latent inter-relationships among entities across multiple sources.

### Performance of the IRES

The performance of our model is evaluated on the annotated abstracts as shown in Table 1.

**Table 1:** Performance Evaluation of the IRES on the annotated abstracts.

Article Type	No. of Abstracts	No. of Relations	Precision	Recall	F1-Score
Abstract	98	354	93%	84%	88%

**Fig 9:**
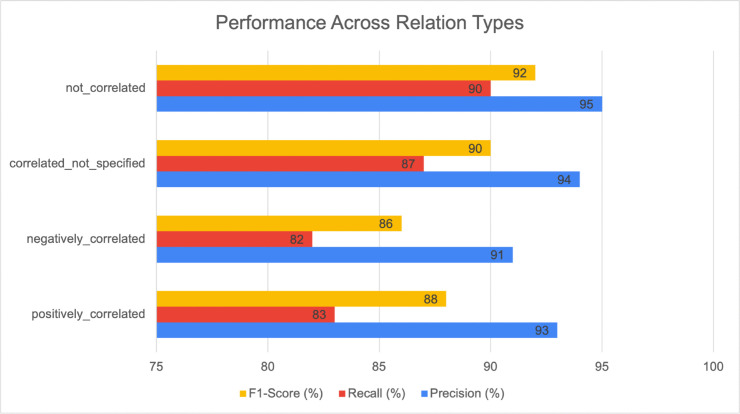
Performance across relation types. Relation-type-specific evaluation of IRES showing precision (%), recall (%), and F1-score (%) for four correlation categories extracted from the annotated abstracts.

To further assess whether the system performs uniformly across different relation types, we conducted a relation-type-specific evaluation, as shown in Fig 9.

This analysis provides a more granular view of how well IRES generalizes across the four major categories: positively_correlated, negatively_correlated, correlated_not_specified, and not_correlated.

## Discussion

In this work, we developed an application that can aid researchers in optimizing glycan profiles for biopharmaceutical manufacturing. Our knowledge graph-based query platform enables researchers to explore relationships among variables critical to therapeutic glycan profile research. The integration of data into a knowledge graph allows analysis of complex dependencies that traditional methods might miss. Through our illustrative examples, we demonstrated the platform’s ability to connect information from diverse sources and reveal implicit links between the critical process parameters and product quality attributes at both term level and Ontology level. Further, our query interface allows users to track the information source of each triple. In addition to displaying the results in a tabular format, the graph display provides an intuitive visualization of the independent facts. However, our knowledge graph interface presents facts extracted from individual sentences without the experimental context presented in the literature. This is primarily designed for sophisticated users such as domain experts in Waters Corporation. Supplementing the triple facts with experimental context from the studies could yield a knowledge graph with richer context-specific semantics and eventually an improved and context-dependent exploration interface which can expand its user space. Currently, our interface is limited to displaying node labels and relationship types without the ability to explore node or edge properties stored in the knowledge graph. The graph display interface could be further enhanced for interactive visualization of node and edge properties.

Our knowledge graph framework systematically integrates text-mined evidence, standardized dictionary concepts, and ontological relationships to uncover both direct and indirect factor-outcome relationships. This approach demonstrates three key advantages: (1) unification of disparate data sources through semantic normalization, (2) structured data representation and governance, and (3) multi-scale analysis enabled by integration with ontology. The resulting system not only reveals established relationships but also infers novel connections through graph traversal across conceptual levels, providing a powerful tool for comprehensive biopharmaceutical manufacturing process analysis. Although not a limitation specific to our study, scaling the graph significantly with new node entities and relationships from additional literature might impact performance.

Our approach of synonym consolidation via dictionary concepts allowed seamless integration of node entities. The ontology developed as part of this study is curated by domain experts. However, our dictionary and ontology concepts are based on the entities extracted from a subset of literature mined for this work and are therefore limited in magnitude. Extending the literature corpus and scaling the knowledge graph with relationships from additional scientific sources requires further manual curation of dictionary and ontology concepts. In future work, we plan to enhance interoperability by aligning our ontology with established standards (e.g., ChEBI (https://www.ebi.ac.uk/chebi/), NCIT (https://www.cancer.gov/research/resources/resource/197), MeSH (https://www.ncbi.nlm.nih.gov/mesh/)) and depositing it on public ontology platforms such as OLS (https://www.ebi.ac.uk/ols4/) and BioPortal (https://bioportal.bioontology.org/) to promote reuse and integration.

While our text-mining evaluation utilized a limited corpus, the achievement of 88% F1-score demonstrates the effectiveness of our rule-based approach for therapeutic glycan research, a domain where annotated data remains scarce. This performance validates our methodology despite current data constraints.

Overall, the system achieved consistent performance across all relation types, with F1-scores ranging between 0.86 and 0.92. The precision remained above 0.91 for all categories, indicating strong reliability in identifying valid relations. The slightly lower recall values (0.82–0.87) reflect occasional misses in longer or syntactically complex sentences. This distribution suggests that IRES is not overly biased toward any specific relation type, demonstrating stable extraction behavior across positive, negative, and unspecified correlation types. Although the evaluation was primarily conducted on abstracts, which typically contain simpler linguistic structures, the model also showed robustness when qualitatively examined on full-text sections.

In several cases, IRES successfully captured complex relations consistent with the annotated corpus, highlighting its potential applicability to more linguistically diverse and information-rich text segments. A careful qualitative inspection of the extracted impact relations from a more linguistically complex section (Results) revealed that our model was able to capture the correct relations that were already annotated in the abstracts.

For example, in [PMID: 31544815] [55] (Results section), the sentence “The reduction of core fucosylation in the Fc-part of antibodies leads to enhanced ADCC and is therefore the aim of several investigations in the literature” produced the extracted relation: **“core fucosylation (Affector)" negatively correlates “ADCC (Affected)"** which is the same relation mentioned in the “Use Cases and Sample Queries" part for another abstract [PMID: 20639190] [40] in Use Case 2, Sentence 2, where the latter instance was annotated. Similarly, we observed consistent extraction in [PMID: 30552760] [41] (Abstract section), which states: “Furthermore, 6.9% increased sialylation was detected through the addition of 30 μ M dexamethasone in combination with the same manganese, uridine, and galactose mixture used to increase total galactosylation.” Here, the relation **“manganese (Affector)" positively correlates “galactosylation (Affected)"** was successfully captured in Use Case 3, Sentence 1, extracted from the Results section [41].

These examples demonstrate that even though we could not conduct a formal evaluation due to the lack of annotated data for full-length articles and book chapters, our text-mining tool was still able to successfully extract biologically meaningful relations from complex sentence structures and extended narrative contexts.

This study also serves as a proof-of-concept illustrating how the integration of text mining and knowledge graph approaches can transform unstructured biopharmaceutical literature into structured, queryable knowledge. We primarily focused on annotated abstracts since they concisely summarize experimental findings and are widely used in biomedical text-mining evaluations. This approach is consistent with prior systems such as RLIMS-P [56], miRTex [57], and eFIP [34], which were developed and validated on annotated abstracts yet demonstrated comparable performance when applied to full-text articles. Although large-scale transformer-based models such as BERT and Large Language Models (LLMs) could further improve relational inference, their effective training requires extensive domain-specific annotations, which are currently limited in the biopharmaceutical context. Hence, our rule-based strategy was chosen to balance interpretability and performance while operating efficiently on a smaller curated dataset.

Although we reviewed two state-of-the-art text-mining systems, including PubTator [58] and BioREx [59], we were not able to conduct a direct performance comparison since these tools are designed for fundamentally different tasks. Both primarily focus on extracting molecular-level relations involving genes, mutations, and chemicals, whereas our framework targets entities central to biopharmaceutical process optimization, such as process parameters (e.g., osmolality, culture conditions), biological processes (e.g., core-fucosylation, enzymatic catalysts), clinical outcomes (e.g., antibody-dependent cell cytotoxicity), and bioreactor conditions (e.g., pH, temperature). These specialized entities are not annotated by PubTator or BioREx and the relationship is also not established in the existing tools. Therefore, a quantitative comparison would not yield meaningful insight. Instead, our evaluation focuses on demonstrating the reliability and interpretability of the rule-based framework within its intended domain.

A limitation of our text-mining approach is the extraction of relationships based on individual sentence contexts without the overall study context. More sophisticated techniques, such as machine-learning-based relation extraction approaches, could aid in extracting contextually relevant relationships. Future improvements could include expanding dictionary concepts, refining the ontological hierarchy, and incorporating more annotated articles to enhance the tool’s robustness. Such improvements would particularly benefit from the creation of a comprehensive, domain-specific dictionary, which is essential for maintaining high precision by excluding irrelevant terms while capturing biologically meaningful relationships.

## Conclusion

This study presents an integrated text-mining and knowledge graph system designed to accelerate optimization of therapeutic glycan profiles. Our framework extracts and structures critical process-parameter relationships with the product quality attributes for glycan profile optimization from scientific literature, transforming unstructured data into actionable knowledge. The system’s core innovation lies in its dual-component architecture: (1) a text mining-based relation extractor that identifies impact relationships with 88% Precision, and (2) a Neo4j knowledge graph that synthesizes these relationships with ontological and dictionary concepts to reveal latent relationships.

The knowledge graph’s strength stems from its semantic organization, ontology-driven hierarchies that provide biological contexts, while dictionary concepts resolve terminological variability, together enabling precise multi-scale queries. Researchers can interactively explore parameter-glycan relationships through an intuitive web interface, with results visualized as both network graphs and structured tables.

While initially validated on glycan optimization, our methodology demonstrates broader potential for biopharmaceutical manufacturing process development. Future enhancements will expand the dictionary coverage, refine ontological relationships, and incorporate machine learning to improve relationship extraction. This work establishes a foundation for data-driven biopharmaceutical manufacturing, where knowledge graphs bridge the gap between published evidence and process optimization decisions.
